# Metal(loid)s in Common Medicinal Plants in a Uranium Mining-Impacted Area in Northwestern New Mexico, USA

**DOI:** 10.3390/plants11152069

**Published:** 2022-08-08

**Authors:** Christine Samuel-Nakamura, Abdul-Mehdi S. Ali

**Affiliations:** 1 School of Nursing, University of California Los Angeles (UCLA), 4-246 Factor Building, Mailcode 691821, Los Angeles, CA 90095, USA; 2 Department of Earth and Planetary Sciences, University of New Mexico, Northrop Hall MSCO 3-2040, Albuquerque, NM 87131, USA

**Keywords:** herbal remedies, Diné reservation, environmental justice, arsenic, cadmium, selenium, ceremonial use, American Indian, *Bouteloua gracilis*, ethnobotanical plants

## Abstract

The objective of this study was to determine uranium (U) and other metal(loid) concentrations (As, Cd, Cs, Pb, Mo, Se, Th, and V) in eight species of plants that are commonly used for medicinal purposes on Diné (Navajo) lands in northwestern New Mexico. The study setting was a prime target for U mining, where more than 500 unreclaimed abandoned U mines and structures remain. The plants were located within 3.2 km of abandoned U mines and structures. Plant biota samples (*N* = 32) and corresponding soil sources were collected. The samples were analyzed using Inductively Coupled Plasma–Mass Spectrometry. In general, the study findings showed that metal(loid)s were concentrated greatest in soil > root > aboveground plant parts, respectively. Several medicinal plant samples were found to exceed the World Health Organization Raw Medicinal Plant Permissible Level for As and Cd; however, using the calculated human intake data, Reference Dietary Intakes, Recommended Dietary Allowances, and tolerable Upper Limits, the levels were not exceeded for those with established food intake or ingestion guidelines. There does not appear to be a dietary food rise of metal(loid) ingestion based solely on the eight medicinal plants examined. Food intake recommendations informed by research are needed for those who may be more sensitive to metal(loid) exposure. Further research is needed to identify research gaps and continued surveillance and monitoring are recommended for mining-impacted communities.

## 1. Introduction

Diverse populations are disproportionately exposed to toxic materials by virtue of proximity [1]. American Indian (AI) communities are at risk for worsened health burdens, which may be compounded by environmental exposures [2]. One-half of the uranium (U) in the United States (US) is found on AI lands, where mining, milling, and processing commonly occur [3], as well as the storage of remaining waste. In the Western US, more than 160,000 abandoned mines exist on or are adjacent to AI homelands [4]. The study setting was a prime target for U mining for military purposes, where northwestern New Mexico (NM) alone contributed 40% of the US U production [5].

Diné (Navajo) lands were one of the prime targets for mining, contributing thirteen million tons of U ore for military use from 1945 to 1988 [6] and leaving more than 550 abandoned and partially unreclaimed U mines, mills, and waste piles [7]. 

The extent of the health impacts on the Diné community exposed to these sites is a public health concern. Uranium enters the body primarily by inhalation or ingestion (via contaminated water or food) and is deposited in tissues, primarily the kidneys and bones [8]. High U exposure studies in mammals have shown kidney chemical toxicity [9]. Uranium and metal(loid)s were examined in this study as they may co-occur environmentally with other metal(loid)s and/or may be associated by way of its decay series. Addressing U and its associated co-contaminant exposures is a challenge for rural communities experiencing a myriad of socioeconomic barriers [10]. Arsenic (As) is a teratogen [11]. Cadmium (Cd) can accumulate in organs and is associated with liver and renal problems [12,13]. Long-term neurodevelopmental, renal, and reproductive problems are associated with lead (Pb) [14]. Toxicosis can occur with high doses of selenium (Se) [15]; semen quality and testosterone have been shown to have an inverse association with molybdenum (Mo) [16,17], which also has a negative effect on renal function [18]. Carcinogenicity has been reported for several metal(loid)s, including As [19,20,21], Cd [19], and Pb [22], whereas others have permanent and/or long-term health sequelae (Mo, Thorium (Th), Vanadium (V), and Cesium (Cs)) [23].

The interaction between humans and plants is known as ethnobotany [24] or rather it is how people of a particular region or culture utilize local (indigenous) plants. It is common knowledge that modern medicines are a direct result of traditional ethnobotanical knowledge and information. Globally, up to 80% of the population relies on traditional medicine for their healthcare needs [14]. Yet, traditional medicines are poorly regulated and monitored in many countries including the US. Medicinal plants are assumed to be safe due to ease of accessibility and availability; they are commonly self-administered or self-prescribed without medical consultation. 

In the US Southwest, there are more than 3000 known plant species, of which the Diné were said to utilize about 450 species for medicinal purposes [25]. In AI communities, local plants are relied upon for their medicinal or healing properties, are consumed as foods/additives, or are relied upon for innumerable cultural purposes. In this community, the primary categories of plant use are medicine, food/beverage, creating dye, paint, ceremonial objects (baskets, paints), and other uses (such as construction, fuel, and implements such as textiles) [25], respectively. Cultural protocol passed down through generations dictates that all parts of the plant should be used without waste, that the plant was selected using strict environmentally sustainable practices (i.e., the strongest and most robust plants are left unharvested to perpetuate the species), and that the harvester has requested permission to use the plant and has practiced thankfulness and respectfulness for its use [26,27]. 

Medicinal plant pharmacological indications, routes, and dosages vary and its administration may include drinking it as a tea or as a concentrated decoction or used in combination with other ingredients (concoction), it may be applied by direct dermal application (as a poultice, salve) and may be inhaled via incense or by steam (sweat bath). Also, plant roots may be directly chewed and ingested (e.g., *Bouteloua gracilis* (Wildenou ex Kunth) Lagasca ex Griffiths). Human exposure can occur through contact with local plant branches, stems, and roots that can be used for cooking and heating (e.g., *Juniperus monosperma* Engelmann), serve as construction materials (e.g., *J. monosperma*), and be used in the creation of numerous cultural implements (baskets, cordage). This could include items integral to traditional healing ceremonies. Plants and their contaminants may be ingested indirectly by humans when locally raised meat (via forage, water, and soil ingestion) is consumed. Phytotherapy self-administration/prescription is commonplace. However, laypersons, herbalists, or traditional practitioners or specialists may provide directions or prescriptions for various indications. A summary of the information on the eight study plant species names/taxonomies, descriptions, and ethnobotanical indications can be found in Table 1. 

The purpose of this study was to determine if eight abundant and readily accessible species of plants, a locally harvested resource on Diné lands in northwestern NM, were contaminated with U and other associated metal(loid)s. Food-chain contamination in locally harvested food in the Diné community in NM was reported as a plausible exposure pathway [42]; harvesting and gathering were found to be common practices [43]. The current study was undertaken to characterize the use of eight common local medicinal plants and contribute novel metal(loid) uptake data. The objective of this study was to compare plant-part concentrations to the World Health Organization (WHO) Raw Medicinal Plant Permissible Level (RMPPL) guidelines and calculate an estimated ingestion risk exposure and compare it to the established food intake guidelines according to the Provisional Tolerable Weekly Intake (PTWI) or Reference Dietary Intake (RDI) or Recommended Dietary Allowance (RDA) and Upper Limit (UL) in eight commonly used medicinal plants in a community impacted by the U mining legacy. 

## 2. Results and Discussion

### 2.1. Data from the Human Harvester Questionnaire

The medicinal plant harvesters (*n* = 6) were evenly divided between genders and the mean (*M* ± *Standard Deviation* (*SD*)) age was 57.25 ± 1.84 (range 53–62). On average, the calculated weekly intake of herbal medicine was 1.17 for ingestion of at least one plant for a mean consumption of 56.5 ± 3.32 years. Per participant reports, plants were located in the wild and did not benefit from artificial watering, soil amendments, or the application of pesticides. All study participants reported sharing the herbs for free with community members on and off Diné tribal lands. No participants reported selling the herbs. The majority of participants self-prescribed and administered the medicinal plants and reported not having consulted a traditional practitioner for their use. Plant harvesting, preparation, storage, and consumption information was passed down from previous generations via elders; they were often laypersons, herbalists, or other traditional practitioners or specialists. 

A study by Tsuji et al. [27] found food-sharing behavior to be common in a North American Indigenous community impacted by mining in a traditional-use territory in Canada. These types of food-sharing behaviors were found to be related to the harvesting of subsistence-type of foods that were found to have a direct exposure impact beyond the mining communities and were considered to be important for assessing and monitoring impacted communities [44], with a special interest in vulnerable groups (children and older tribal members) [45]. Using Geographic Information System (GIS) mapping, the above study [27] demonstrated that longstanding harvesting areas overlapped significantly with contaminated areas and that several important potential routes of exposure were identified and characterized (e.g., ingestion of contaminated foods and drinking water). Using GIS, the current study demonstrated an overlap of medicinal plant gathering and harvesting areas in proximity to mining sites and features; overlap was commonplace and samples fell within a 3.2 km buffer zone of high-risk areas. Proximity (median 3.54 km ((IQR 1.81–8.0)) to U mine and milling sites was found to be a potential contributor to cardiovascular disease [46] in a local GIS study.

### 2.2. Medicinal Plant Parts 

Twenty-seven percent of the medicinal plant species in the study areas consisted of the *B. gracilis* plant, with 12% each of *A. hymenoides*, *A. purpurea*, *P. smithii, P. jamesii,* and *S. cryptandrus* and 6% each of *J. monosperma* and *A. tridentate* plants. The availability and distribution of the sampled plants were representative of the local flora reported in the literature [29,31,32,34,35,36,37,39]. The majority of the plants had greater concentrations in their aboveground parts than their roots. The metal(loid)s that met statistical significance (*p* < 0.05) were Cd, Se, Th, and U (Table 2). The largest metal(loid) concentration differences were found between the aboveground *A. purpurea* plant and its roots for Se (3.50 mg/kg vs. 2.31 mg/kg) and the *P. jamesii* plant and its roots (3.69 mg/kg vs. 2.41 mg/kg). Others that differed by more than 1 mg/kg were *A. tridentate* (2.67 mg/kg vs. 1.55 mg/kg) and *S. cryptandrus* (3.36 mg/kg vs. 2.28 mg/kg). 

In general, comparable results were found (or lower plant concentrations) with herbal plant metal(loid) levels in the study area [42,48], including international studies [49,50,51,52], except there were higher concentrations found with a Th [52] plant study (Table 3). Forage grasses reported for U ranged from 0.5 to 7.7 mg/kg (U in root *M* = 5.0 mg/kg and grass blades 2.4 mg/kg) [42] (Table 3). The plant species reported by local and international studies were dissimilar to the plants reported in this study. Shi et al. [53] reported that various plants are prone to concentrating contaminants in their main roots as they seem to function as a buffer to the aboveground parts of the plant. Similarly, Anke et al. and Soudek et al. found that there were greater metal(loid) concentrations in the plant roots than in the above-ground portions [54,55]; this was particular to U [55]. The uptake of metal(loid)s appeared to differ between species of plants [49,50,51,52]. 

### 2.3. Soil

In most instances, the study findings showed that metal(loid)s concentrated greatest in soil > root > aboveground plant parts, respectively (Table 2). Vanadium was the only metal that exceeded the concentration range of 15 mg/kg. Those metal(loid)s that fell between 10 and 15 mg/kg were Pb, Th, and As and of that, less than 5 mg/kg were Se, Cs, Mo, U, and Cd. The mean soil pH was weakly acidic to neutral in reaction (6.91 ± 0.97). Statistical significance was found in comparing the soil to the aboveground plant parts (soil > plants): V (*p* < 0.001), As (*p* < 0.05), Cs (*p* < 0.05), Pb (*p* < 0.05), Mo (*p* < 0.05), Th (*p* < 0.05), and U (*p* < 0.05). The soil concentrations were greater than the plant roots for all sampled plants: As, Cs, Pb, Mo, and Th (*p* < 0.001), V (*p* < 0.01), U, Se, and Cd (*p* < 0.05). These findings were similar to local and international plant studies examining different species of medicinal plants for metal(loid) content (As, Cd, Cs, Pb, Mo, Se, Th, U, V [43,48], Cd, and Pb [50] (Table 3)). In a regional tea soil study [43,48], there were comparable results for As, Cd, Cs, and V but greater concentrations of Pb, Se, and U; there were smaller concentrations of Mo and Th (Table 3). Regional plant and soil studies were also conducted for a different species of herbal plant (*T. megapotamicum*). A local study found comparable concentrations of Se in high-impact soil areas [56] but greater U soil concentrations were found in non-control areas [42,57].

The soil pH was comparable to other locally harvested plant and soil studies; they ranged from 6.3 to 6.5 (herbal tea and squash studies) [43,48]. More acidic soils have been demonstrated to increase the transfer and uptake of various metals such as Cd [58] and were thought to increase the likelihood of co-occurrence with other metal(loid)s, which also seems to be dependent on the physiochemical make-up of the soil and individual uptake of metals by various plant species [55,59,60]. It was beyond the scope of this study to describe all variables associated with the uptake of metal(loid)s in herbs from the soil. 

For the herb-harvesting activities and consumption reported in this study, the main exposure to metal(loid)s appears to be via soil. In general, the current study has demonstrated that soil contained the greatest amounts of metal(loid)s compared to plant part samples (Table 2), which is comparable to local tea [43], vegetation [42], and crop studies [48]. 

### 2.4. WHO RMPPL

The soil Cd concentration levels were exceeded for the WHO RMPPL of 0.3 mg/kg by 2.9 times for the aboveground plant parts for *P. jamesii (M =* 0.87 ± 1.42 mg/kg, Table 2). Five aboveground plant parts (*A. hymenoides: M =* 1.31 ± 0.19 mg/kg; *A. purpurea: M* = 1.22 ± 0.32 mg/kg; *B. gracilis*: *M* = 1.08 ± 0.47 mg/kg; *P. smithii*: *M* = 1.19 ± 0.41 mg/kg; and *P. jamesii*: *M* = 1.40 ± 0.28 mg/kg) exceeded the As concentration level of 1 mg/kg for the WHO RMPPL [47]. Of all the plant species sampled, study participants reported consuming *B. gracilis* root *(M* = 1.16 ± 0.41 mg/kg) for medicinal purposes; this was found to have exceeded the As WHO RMPPL by more than 3.5 times the recommended level. There were no exceedances for Pb WHO levels (10 mg/kg) for all eight species of plants [47]. 

The WHO RMPPLs were put in place to evaluate the presence of metals in herbal tea formulations and tinctures [47]. There are no permissible levels for Cs, Mo, Se, Th, U, and V.

A local herbal plant study found that the WHO RMPPL was exceeded for Cd in a popular species of tea, *T. megapotamicum* (*M* = 0.35 ± 0.31 mg/kg) and was higher in high-vehicular-traffic areas (*M* = 0.68 ± 0.11 mg/kg; *p* < 0.001) than low-traffic areas (*M* = 0.10 ± 0.06 mg/kg; *p* < 0.001) [43]. International medicinal plant studies did not find PTWI exceedances in other species of plants [61,62]. 

### 2.5. Human Intake Calculations for As, Cd, and Pb

The weekly intake calculations for As for each plant ranged from 0.29 to 0.82 μg/kg, 0.02 to 0.51 μg/kg for Cd, and 0.29 to 1.55 μg/kg for Pb (Table 4). Collectively, the PTWI percentages were low and fell below 7.3% (range 0.29–7.3%) of the weekly intake for all plants examined. 

The PTWI limits are 15 μg/kg body weight (BW), 7 μg/kg BW, and 25 μg/kg BW for As, Cd, and Pb, respectively [63,64]. There are currently no PTWI guidelines set for Cs, Mo, Se, Th, U, or V. All metal(loid) PTWI levels were below the level of concern for all plants examined. The PTWIs reported here were generally lower than those reported for squash and herbal tea plants [43,48] in a comparable regional study.

### 2.6. Human Intake Calculations for Mo, Se, and V

The daily intake calculation for Mo ranged from 0.28 to 0.91 μg, 0.92 to 2.01 μg for Se, and 0.22 to 4.86 μg for V (Table 5). The percentages for the RDA and RDI all fell below 3.7% for each plant studied. The UL percentages were considerably lower and did not exceed 0.5% for all medicinal plants sampled. 

For Mo, the RDA is 45 μg/day with a tolerable Upper Limit (UL) of 2000 μg [65]. The RDI for Se for adults is 55 μg/day with a tolerable UL of 400 μg/day [66]. The UL for V is 1800 μg/day but there are no RDA or RDI guidelines [65]. There are no set RDIs/RDAs for As, Cs, Pb, Th, U, or V. There are no UL guidelines for As, Cd, Pb, Th, or U. In a local study area report, the RDAs, RDIs, and ULs were lower than those reported for squash and herbal tea biota [43,48]. The calculated RDIs/RDAs for Mo, Se, and V were small; however, these may not be completely reflective of the overall diet. It is likely that the Mo, Se, and V RDIs/RDAs were met by the consumption of other foods in the regular overall diet. This study only focused on a small portion of the entire food intake. For this cohort, supplemental Se and Mo in the diet may be needed (if not met by the regular overall diet) and is available in foods such as meat, legumes, grains (Se), and nuts (Mo) [65]. The advice of a dietitian and healthcare provider is recommended for any dietary changes in similar settings.

### 2.7. Human Implications for Intake Calculations

The intake estimates demonstrate that the consumption of each herbal medicine individually may not be of concern in the current cohort with an intake of 1.17 times per week. Upon direct comparison to the WHO RPPML, several plant species’ concentration levels were found to exceed the permissible levels for As and Cd. When the calculation incorporated a reference to body weight (60 kg) for the cohorts’ weekly intake (1.17 times a week), the PTWI (As, Cd, and Pb), RDAs/RDIs (Mo and Se), and ULs (Mo, Se, and V) were not exceeded for all eight species of medicinal plants. The former guidelines are based on Acceptable Daily Intake and the latter guidelines are more appropriate for long-term or chronic exposure to metal(loid)s [63]. More recent recommendations by the WHO [67] support the use of PTWI for measuring accurate medicinal plant material metal(loid) intake exposure. In this study cohort, participants reported extensive years of exposure to medicinal plant harvesting consumption (56.5 ± 3.32) as well as participation in other related outdoor harvesting activities. This provides support for the use of the PTWI guidelines as an accurate measure of chronic or long-term exposure. 

For this study, we only reported individual plant concentration intake estimates consumed on a weekly basis and examined only a portion of the overall dietary intake. In some instances, there was a potential for guideline exceedances if several medicinal plants were used in mixtures or consumed on a more frequent basis. Further, if study participants were consuming additional locally raised and harvested foods (including local water) the combination may exceed the estimates reported here. For a more accurate intake estimate, collective food intake assessments that examine all aspects of one’s dietary intake are recommended. It was beyond the scope of this study to report the estimates of all conceivable mixtures of phytotherapies or to consider every route of administration. In most study case scenarios, medicinal herbs were typically consumed for short periods or were reserved specifically for special albeit infrequent curative ceremonies and their over-consumption was uncommon. Lastly, examining metal(loid) uptake and their calculated intake from other medicinal plants that were not examined in this study is warranted.

This population group has disproportionately high rates of hypertension, diabetes, cancer, cardiovascular disease [2,46], renal disease, and other comorbidities [68]. Metal(loid) exposures are known to worsen these comorbidities. Further, there is little research on the collective bioeffects of co-occurrent contaminants. More research is needed for high-risk groups as they may be more susceptible to the effects of metal(loid)s. High-risk groups include the very young, lactating or pregnant women, older adults, and those with cardiac, renal, and immune function problems. The level to which exposure is a danger to high-risk persons and other interrelated factors are unknown and need further investigation. It is recommended for individuals that consume traditional medicinal plants to consult with their healthcare provider when consuming alternative therapies to avoid untoward medication interactions. 

### 2.8. Limitations

There were several study limitations. There was ample literature documenting the indications for various medicinal plant remedies in this community; however, there was significantly less documentation in relation to dosage information. Several sources of information were available documenting the use of various medicinal plants during pregnancy and the postpartum period [28,33] and for the treatment of infants and children [28,37], but for all age groups, there was no dosage information available. As there was scant dosage information to glean for the study calculations, we relied upon comparable studies. For instance, we provided an estimate of oral intake by using the equivalency of one cup of tea containing one g of plant material [61,69]. Also, exposure in terms of routes of administration was not examined in this study due to the lack of detailed pharmacological information. For example, inhalation (via incense (e.g., *P. smithii, A. tridentate*) or sweat bath steam or other exposures by smoke or mist or aerosolization) and dermal exposure (e.g., *B. gracilis*, *A. tridentate, J. Monosperma*) were not calculated for this report. Future examination is needed to establish dosages and to include various routes of administration such as inhalation and dermal skin exposures.

Other locally derived environmental sources of exposure may add or compound the risks. For instance, it is common practice for people to use local water (regulated and unregulated) to steep the teas or medicinal concoctions possibly using a suite of plant mixtures. Further, such plant mixtures introduce several complexities; without detailed pharmacologic information, synergistic, additive, and antagonistic effects are difficult to determine. In fact, some studies have identified that metal(oid)s may dissociate in water at certain water temperatures [70,71] and that the pH of infusion water may be a factor in uptake [61,72]. These factors warrant further investigation.

A plausible reason for the lack of dosage-specific or other detailed phytotherapy information may be that some tribal communities are protective of this information. Researchers and other experts have reported a general reluctance by tribal members or informants to report on healing ceremonies/medicinal plants as this knowledge is seen as sacred and such esoteric medicinal and ceremonial knowledge is exclusively for the dispensation/treatment/handling by Diné medicine-people with extensive training or who have undertaken apprenticeships [28]. For this paper, the researchers have not reported any new information in this study on medicinal plant indications (including references to specific ceremony names) that has not been published elsewhere [28,29,31,32,33,34,35,36,37,38,39,40,41]. It is rather an organized compilation and report of existing medicinal plant information published by researchers in direct consultation with expert Diné informants [28,29,31,32,33,34,35,36,37,38,39,40,41] who are vetted specialists such as herbalists, medicine-people, or healers. The resource is meant to be a reference source for researchers, healthcare providers, traditional medicine healers, and tribal community members and leaders. Further, it is not the purpose of this paper to report on pharmacokinetics but rather to inform and identify knowledge gaps in this area of medicinal plant research. Pharmacological intake, absorption, bioavailability, distribution, metabolism, and excretion are very complex processes and require extensive research. Further, there are many complicated individualistic (e.g., age, chronic and acute health problems, metabolism, genetics, diet/nutritional status, etc.) and interrelated environmental variables to be considered.

## 3. Materials and Methods

This was a descriptive, comparative study examining contamination levels in locally harvested medicinal plants and soils from reservation areas within a 3.2 km radius of previously U-mined and disrupted areas. Data obtained from the Diné Network for Environmental Health (DiNEH) study cohort [42] served as one of the sources for identifying the subjects and samples of food, herbs, water, and soil. Additional participants were recruited using snowball methods (word-of-mouth), home visits, and advertising at public tribal community events. Of the DiNEH cohort [42] respondents, those individuals who reported harvesting plant foods were recruited for participation in the present study. Plant biota were selected based on active use by study participants and their proximity to mining structures. The medicinal plant data were compared and reported to reflect an accurate estimated measure of metal(loid) intake in humans via medicinal plant ingestion.

### 3.1. Study Setting

This study was reviewed and approved by the dual Institutional Review Board (IRB)s, the Navajo Nation Human Research Review Board, and the University of California, Los Angeles, (UCLA) IRB. The eight species of plants identified in this study are not listed as endangered or threatened according to the Navajo Nation Department of Natural Resources [73] and the NM Energy, Minerals, and Natural Resources Department [74].

**Figure 1:**
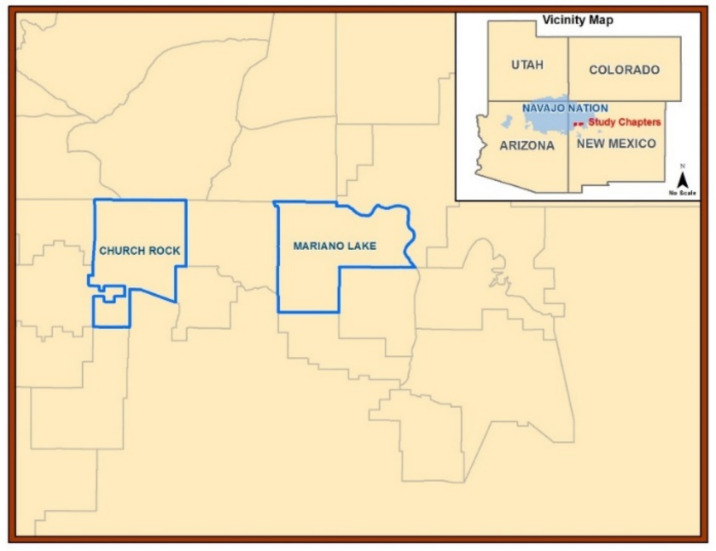
Research area map. Cartographic map of the Navajo Nation in the Four Corners region of the US Southwest. New Mexico communities or “Chapters”: Churchrock (land mass 233 km^2^) and Mariano Lake (land mass 272 km^2^) provided biota and soil samples.

The field research area is a semi-arid to arid region of the US Southwest in northwestern NM on Diné reservation lands (Figure 1). The average precipitation was found to be <25 cm per year according to meteorological data for NM (Western Regional Climate Center Western US Climatic Historic Summaries) during the study period. The Mariano Lake Chapter is 272 km^2^ of land mass and the Churchrock Chapter is 233 km^2^ (total land mass of 505 km^2^). Recruitment was initiated in May 2012 and enrollment began in July 2012. All samples were collected from 10 November to 13 December 2012. This study focused on locally harvested plant biota and was part of a larger research project that examined subsistence farming on the reservation, including the metal(loid) contamination of herbs, sheep, crops, and associated data [43,48].

### 3.2. Human Harvester Questionnaire Data

The Diné Plant-Animal-Human-Questionnaire was administered to collect demographic information and collect overall local food harvesting data. Information on specific harvesting exposure activities was obtained. The Diné Wild Plant/Herb Intake Questionnaire was used to collect information on herbal plant harvesting and consumption. Data collected included plant use; indications; the amount, frequency, and duration of consumption; incidences and the extent of herb sharing and sales; relevant cultural uses for the medicinal plants; and traditional practitioner information.

### 3.3. Plant Identification and Nomenclature

Live parallel plants were collected, dried, and pressed for identification and archival. A plant collection description log was collected. Color photographs were taken of each plant. The University of New Mexico (UNM) Herbarium identified and archived the plant samples. Global Positioning System (GPS) instrumentation (Trimble Navigation Limited, Westminster, CO, USA) was utilized to collect location data and conduct spatial proximity analysis. Data differential correction was completed within 72 h of data capture using Pathfinder Office version 5.30 (Trimble Navigation Limited, Westminster, CO, USA).

### 3.4. Medicinal Plant Samples 

Eight species of medicinal plants were collected and identified. Live medicinal plant samples were collected from wild, non-cultivated sources within a 3.2 km radius of the central part of abandoned U mines and features (mine portals, pits, rim strips, vertical mine shafts, and prospect areas). The above-ground portions and roots of live plants were stored in polyethylene (PE) plastic Ziplock^®^ bags. The plant samples were photographed, weighed, bagged, and placed on dry ice for shipment for analysis by the UNM Analytical Chemistry Laboratory Earth and Planetary Sciences Department. The medicinal plant flowers, leaves, stems, and roots were analyzed for metal(loid)s (As, Cd, Cs, Pb, Mo, Se, T, U, and V) using inductively coupled plasma–mass spectrometry (ICP-MS).

### 3.5. Soil Samples 

For each medicinal plant sample, parallel soil samples were collected. To avoid cross-contamination, a silicon-coated core sampler (Art’s Manufacturing and Supply Inc. (AMS), American Falls, ID, USA) was utilized. A slide hammer with a stainless-steel hand auger was employed to collect soil samples using a PE liner (AMS Core Sampling Mini-kit, American Falls, ID, USA). One hundred grams (g) of soil were collected for each plant from a 0–25 cm depth. The soil samples were analyzed for metal(loid)s (As, Cd, Cs, Pb, Mo, Se, Th, U, and V) using ICP-MS.

### 3.6. Sample Analysis

Medicinal plant and environmental sample preparation and analysis are reported in detail in previous publications [43,48]. The biota and soil samples were stored in a −20 °C freezer before preparation and analyses. The organic plant samples were first washed thoroughly with 18 mega Ohm water to remove any suspended materials on the plants’ surfaces. In addition, the samples were then soaked in a very dilute solution (0.001 M HCl) to ensure the removal of clay particles and any pollutants on the plants’ surfaces. The samples were then oven dried at 65 °C until the samples’ weight stabilized. The samples were prepared by weighing 2 g of dry mass into the digestion tube. Two mL of Hydrogen Peroxide (H_2_O_2_) and 5 mL of ultra-high purity nitric acid (HNO_3_) were added, and the solid plant and soil samples were gradually heated to 95 °C and digested for two hours. The digested samples were transferred into 50 mL volumetric flasks and brought to volume using 18 mega Ohm water. Three mL of HNO_3_ (reagent blank) was run with each batch of samples. 

A PerkinElmer NexION 300D ICP–MS (Waltham, MA, USA) coupled with an ESI SeaFast SP3 auto-sampler were used to analyze the digested samples in both direct (Anhydrous Ammonia for trace metals) and hydride (Oxygen for Arsenic) modes to significantly minimize mass interferences. The instrument detection limits are as follows: As 0.3 μg/L, Cd 0.1 μg/L, Mo 0.02 μg/L, Pb, 0.008 μg/L, Se 1.3 μg/L, and U 0.008 μg/L. 

For each sample, three replicates (including Certified Reference Materials (CRMs)) were measured. Certified National Institute of Standards and Technology (NIST) Standard Reference Materials were used and include: 2709 San Joaquin soil (NIST, Gaithersburg, MD, USA) and 1573a (tomato leaves, NIST Gaithersburg, MD, USA) and yielded the following values: for Cd 1.474 ± 0.11 mg/kg (1.52 ± 0.04 mg/kg (CRM tomato leaves)) and V 0.94 ± 0.07 mg/kg (0.84 ± 0.01 mg/kg (CRM tomato leaves)) and Cd 0.64 ± 0.09 mg/kg (0.37 ± 0.02 mg/kg (CRM soil value)) and V 83.2 ± 7.7 mg/kg (110 ± 11 mg/kg (CRM soil value)). The relative standard deviation was found to be within the range of 7.1–13.8%.

### 3.7. Provisionable Tolerable Weekly Intake (PTWI) Calculation Equation

The metal(loid) PTWI calculations were derived by utilizing this equation [61,68]:

PTWI = daily intake of metal(loid)s = ∑[concentration of metal(loid) in herb × mean of herbal intake (grams per person per day)]; weekly intake of metal(loid)s = daily intake × seven days a week;  weekly intake per body weight (kg) (PTWIs) = weekly intake or reference body weight (60 kg).  Consumption based on the number of grams per day that herbal medicines were consumed (5 g) based on comparable data. (1)

### 3.8. Statistical Analysis

Statistical analysis was undertaken to utilize the Statistical Package for the Social Sciences (SPSS) for Windows (version 28, IBM, Armonk, NY, USA). Metal(loid) concentration levels in the medicinal plants and corresponding soil samples were reported as milligrams per kilogram (mg/kg). The summary data included means, standard deviations, medians, ranges, and percentages. The differences between the metal(loid) levels in the medicinal plant parts and soil were compared, with significance determined by Student’s *t*-tests. A *p*-value of <0.05 was considered significant. The absolute value of the *t*-statistic was reported along with the relevant means and the interpretation of the directions of differences.

## 4. Conclusions

The WHO RMPPLs were exceeded for As for five aboveground plant parts (*A. hymenoides*, *A. purpurea*, *B. gracilis* (including plant root), *P. smithii*, *P. jamesii*) and two plant roots for Cd (*P. jamesii* and *A. Tridentate*); however, when the PTWI were calculated using the study participant intake data, all plant concentrations fell below the level of concern for metal(loid)s that have established food intake guidelines. There are no established intake guidelines for Cs, U, and Th. The current data do not appear to demonstrate a risk of metal(loid) ingestion above the average ingestion intake in this study cohort for the eight species of medicinal plants examined. Further study is needed to address the study limitations and the identified research gaps. The limitations to be addressed include further characterizations of medicinal plant dosages, indications and administration routes, and the health effects on high-risk groups. Continued research, surveillance, and monitoring are needed in uranium mining-impacted communities.

## Figures and Tables

**Table 1:** Plant names (scientific, common, and Diné names), biota description/distribution, and ethnobotanical indications.

Plant Names	Description/Distribution	Ethnobotanical Indications
*Achnaterum hymenoides* (Roemer & Schultes) Barkworth Indian ricegrass Diné name: “*Nididlídii” “scorched”* [28] (p. 102)	A tufted bunch grass with narrow blades about 53 cm tall. This plant is primarily located in sand dunes and its elevation range is 600-3000 m [29]. It is found in the foothills, plains, and (inter)mountain basins of the western US [29].	This plant was a food source for the early Diné and was cooked as mush, bread dumplings, and cakes [30]. It served as a material for clothing and bedding for the early Diné [28]. In the present day, it is an important feed source for animals and livestock [29].
*Aristida purpurea* Nuttall Purple threeawn Diné name: “*Dl*ǫ’ǫ’ *’* *bibé’ézhóó’*“ “prairie dog comb” [28] (p. 128)	This is a short (<4.4 cm) perennial bunch grass with fine curly leaves. It grows in elevations less than about 2000 m [28]. This grass is widely distributed throughout Canada, western North America, and Mexico [31].	This plant is a ceremonial ^a^ medicine and may often be combined with other mixtures of plants for therapy [28].
*Artemisia tridentate* Nuttal Big sagebrush Diné name: “*Ts’ah”* “the sagebrush” [28] (p. 106)	Gray-green-foliaged aromatic shrub that grows to heights of about 1.8 m. It has a woody stalk and flowers in late August through early October. The range of growth is from elevations of between 1500 and 2000 m [28]. This shrub has a vast distribution in British Columbia, Baja California, and the eastern Dakotas [32].	When combined with other sagebrush species it is used to treat headaches. As a tea, it is prescribed for postpartum hemorrhage/pain [33], indigestion, and constipation. The stems and leaves are boiled to treat fever, colds, and tuberculosis. It is used for fasting and as a poultice for swelling and foot corns. It is a vital component of several Diné ceremonies^a^ [28,33]. It serves as a fire-starting implement (a ceremonial fire drill) and is a common sweat bath medicine, food, beverage, and medicine [25]. The sagebrush leaves are used to purify (smudge) [25]. It is a universal tonic and is used for swelling and snakebite and implement dye [33]. It is considered a “life medicine” and has special healing powers [25] (p. 42). A poultice can be applied to animal wounds [28].
*Bouteloua gracilis* Blue grama Diné name: “*Tł’oh nástasí*“ “bent grass” [28] (p. 45)	This is a perennial grass that rarely exceeds 61 cm in height and grows in areas up to 2500 m [28]. It has a comb-like spike and grows from June to November and flowers from July to October. It is the most prolific grass on Diné lands. It is found in the Great Plains and the southwestern US, Mexico, and the Canadian Provinces [34].	This plant can be applied to heal cuts on humans and animals or by placing a chewed root directly on the wound. As a tea concoction, it is used for postpartum pain [28]. The plant is used in several cultural ceremonies ^a^ [28]. The plant is an important and vital forage for the local animals and livestock [34].
*Juniperus monosperma* One-seed juniper Diné name: “*Gad biką’ígíí”* “male juniper” [28] (p. 55)	This is a perennial shrub tree. It is found in Oklahoma, Kansas, the US Southwest, and Texas [35].	This medicinal plant is an emetic, used to treat headaches, influenza, abdominal pain, nausea, and as an antihelminth [25]. It is also used to treat acne, arachnid bites, and postpartum pain [28]. For ceremonies ^a^, it is used as an emetic and as a healing implement [28]. Juniper berries can be eaten in the fall and serve as culinary ash (providing sources of iron, zinc, calcium, and potassium) in blue corn dishes. It is a valuable fuel source for heating the home and cooking and its branches and twigs serve as construction materials. Juniper berry tea and twig tea are medicinal. It is used to dye wool and other cultural implements [25].
*Pascopyrum smithii* (Rydeberg) Löve Western wheatgrass Diné name: “*Tl’oh nitl’iz*í“ “brittle grass” [28] (p. 132)	This is a blue-green or pale gray bunch grass that has underground stems and long-living, extensive, strong root systems. It can grow up to 61 cm and grows in patches in elevation ranges of 1200–2500 m [28]. It is found in the soils of the US Southwest, intermountain areas of the western US, and the Great Plains [36].	It is used as incense for various ceremonies ^a^ [28].
*Pleuraphis jamesii* Torrey Galleta Diné name: “*Tł’oh łíchí’í*“ “red grass) [28] (p. 39)	This is a perennial grass with rhizomes and grows in patches to a height of less than 61 cm. It is the second most abundant grass on Diné lands and grows in areas greater than 2000 m [28]. This plant is widely distributed in southern California, Colorado, the desert mountains of Arizona, Nevada, New Mexico, Utah, west Texas, and southern Wyoming [37].	Tea is boiled and given to infants so they “will be strong adults” [28] (p. 39). It is a dietary supplement for children [37] It is an important source of feed for local animals/livestock [37].
*Sporobolus cryptandrus* (Torrey) A. Gray Sand dropseed Diné name: “*Tl’oh*-stoz-ee“ “slender grass” [38] (p. 777)	This grass matures by late May or June [25]. The plant has narrow tightly rolled leaves with a lacy appearance [25]. It is a native plant found throughout North America in the rangelands of the US Southwest and parts of Idaho and Oregon [39].	A food source for local Native peoples in the Four Corners region as a “hot grain cereal”, bread [25] (p. 195), and medicine [30,40]. It is used for ceremonies ^a^ [41] (p. 17).

Note: ^a^ Refer to the listed citation source(s) for specific ceremony names.

**Table 2:** Concentrations of Arsenic, Cadmium, Cesium, Lead, Molybdenum, Selenium, Thorium, Uranium, and Vanadium in eight species of medicinal plants and soil (*Mean* ± *SD* mg/kg, Range).

Plant Species Scientific and Common Name	As mg/kg	Cd mg/kg	Cs mg/kg	Pb mg/Kg	Mo mg/kg	Se mg/kg	Th mg/kg	U mg/kg	V mg/kg
*Achnaterum hymenoides* Indian ricegrass (*n* = 4)	1.31 ± 0.19 1.05–1.50 ^2^	0.07 ± 0.02 0.05–0.10 ^6^	0.39 ± 0.96 0.25–0.45 ^2^	1.86 ± 0.35 1.39–2.22 ^2^	0.73 ± 0.51 0.28–1.29 ^2^	1.32 ± 0.46 0.87–1.95 ^6^	1.23 ± 0.28 1.02–1.63 ^2, 6^	0.43 ± 0.13 0.29–0.58 ^2, 6^	4.27 ± 0.66 3.32–4.84 ^1^
*Achnaterum hymenoides* Indian ricegrass root (*n* = 4)	1.44 ± 0.08 1.39–1.50 ^5^	0.06 ± 0.02 0.05–0.07 ^3, 6^	0.44 ± 0.01 0.43–0.45 ^5^	2.08 ± 0.20 1.95–2.22 ^5^	0.30 ± 0.04 0.28–0.33 ^5^	1.62 ± 0.47 1.28–1.95 ^3, 6^	1.32 ± 0.43 1.02–1.63 ^5, 6^	0.53 ± 0.08 0.47– 0.58 ^3, 6^	4.68 ± 0.22 4.52–4.84 ^4^
*Aristida purpurea* Purple threeawn (*n* = 4)	1.22 ± 0.32 1.02–1.70 ^2^	0.22 ± 0.10 0.14–0.35 ^6^	0.67 ± 0.26 0.46–0.98 ^2^	2.66 ± 0.65 1.96–3.41 ^2^	0.76 ± 0.17 0.66–1.01 ^2^	2.31 ± 1.39 1.09–3.78 ^6^	1.05 ± 0.55 0.31–1.63 ^2, 6^	0.37 ± 0.07 0.28–0.44 ^2, 6^	5.82 ± 1.03 4.61–6.89 ^1^
*Aristida purpurea* Purple threeawn root (*n* = 4)	1.36 ± 0.48 1.02–1.70 ^5^	0.29 ± 0.07 0.24–0.35 ^3, 6^	0.89 ± 0.13 0.79–0.98 ^5^	3.18 ± 0.33 2.95–3.41 ^5^	0.66 ± 0.01 0.66–0.67 ^5^	3.50 ± 0.40 3.21–3.78 ^3, 6^	0.97 ± 0.93 0.31–1.63 ^5, 6^	0.42 ± 0.02 0.41–0.44 ^3, 6^	6.66 ± 0.33 6.42–6.89 ^4^
*Bouteloua gracilis* Blue grama (*n* = 9)	1.08 ± 0.47 0.19–1.96 ^2^	0.10 ± 0.06 0.00–0.22 ^6^	0.35 ± 0.12 0.11–0.53 ^2^	1.98 ± 0.66 0.63–2.99 ^2^	0.77 ± 0.44 0.24–1.65 ^2^	1.76 ± 0.60 0.81–2.99 ^6^	1.04 ± 0.70 0.36–3.26 ^2, 6^	0.16 ± 0.66 0.06–0.25 ^2, 6^	1.54 ± 0.34 0.00–5.69 ^1^
*Bouteloua gracilis* Blue grama root (*n* = 9)	1.16 ± 0.41 0.38–1.58 ^5^	0.12 ± 0.06 0.03–0.22 ^3, 6^	0.37 ± 0.11 0.17–0.53 ^5^	2.15 ± 0.62 0.98–2.85 ^5^	0.60 ± 0.49 0.24–1.65 ^5^	1.79 ± 0.58 1.02–2.83 ^3, 6^	1.23 ± 0.86 0.49–3.26 ^5, 6^	0.18 ± 0.05 0.12–0.25 ^3, 6^	3.51 ± 1.70 0.01–5.68 ^4^
*Pascopyrum smithii* Western wheatgrass (*n* = 4)	1.19 ± 0.41 0.81–1.62 ^2^	0.08 ± 0.04 0.04–0.11 ^6^	0.41 ± 0.18 0.25–0.58 ^2^	1.93 ± 0.91 1.13–2.76 ^2^	0.33 ± 0.47 0.28–0.39 ^2^	1.16 ± 0.34 0.86–1.54 ^6^	1.04 ± 0.61 0.51–1.78 ^2, 6^	0.57 ± 0.42 0.20–1.08 ^2, 6^	3.45 ± 1.58 2.02–4.89 ^1^
*Pascopyrum smithii* Western wheatgrass root (*n* = 4)	1.53 ± 0.12 1.45–1.62 ^5^	0.11 ± 0.00 0.11–0.11 ^3, 6^	0.57 ± 0.01 0.56–0.58 ^5^	2.72 ± 0.05 2.69–2.76 ^5^	0.30 ± 0.03 0.28–0.33 ^5^	1.45 ± 0.12 1.37–1.54 ^3, 6^	1.55 ± 0.33 1.31–1.78 ^5, 6^	0.92 ± 0.23 0.73–1.08 ^3, 6^	4.81 ± 0.11 4.73–4.89 ^4^
*Pleuraphis jamesii* Galleta (*n* = 4)	1.40 ± 0.28 1.06–1.75 ^2^	0.87 ± 1.42 0.12–3.00 ^6^	0.34 ± 0.06 0.32–0.45 ^2^	2.15 ± 0.14 2.05–2.36 ^2^	1.07 ± 0.48 0.53–1.44 ^2^	2.41 ± 1.77 0.02–4.16 ^6^	0.61 ± 0.37 0.18–0.82 ^2, 6^	0.12 ± 0.04 0.11–0.14 ^2, 6^	3.89 ± 0.39 3.62–4.45 ^1^
*Pleuraphis jamesii* Galleta root (*n* = 4)	1.41 ± 0.49 1.06–1.75 ^5^	1.58 ± 2.00 0.21–0.22 ^3, 6^	0.44 ± 0.02 0.42–0.45 ^5^	2.21 ± 0.23 2.05–2.36 ^5^	1.34 ± 0.14 1.24–1.44 ^5^	3.69 ± 0.67 1.37–1.54 ^6^	0.82 ± 0.01 0.81–0.82 ^5, 6^	0.13 ± 0.02 0.11–0.14 ^3, 6^	4.03 ± 0.59 3.62–4.45 ^4^
*Sporobolus cryptandrus* Sand dropseed (*n* = 4)	0.85 ± 0.26 0.55–1.12 ^2^	0.19 ± 0.03 0.16–0.22 ^6^	0.54 ± 0.21 0.35–0.75 ^2^	2.00 ± 0.73 1.36–2.72 ^2^	1.09 ± 0.26 0.82–1.33 ^2^	2.28 ± 2.23 0.22–5.30 ^6^	1.50 ± 1.30 0.41–3.07 ^2, 6^	0.17 ± 0.08 0.10–0.26 ^2, 6^	4.35 ± 1.52 2.90–6.03 ^1^
*Sporobolus cryptandrus* Sand dropseed root (*n* = 4)	1.06 ± 0.08 1.00–1.12 ^5^	0.22 ± 0.01 0.21–0.22 ^3, 6^	0.72 ± 0.04 0.69–0.75 ^5^	2.62 ± 0.15 2.51–2.72 ^5^	1.08 ± 0.00 0.82–1.33 ^5^	3.36 ± 2.74 3.21–4.16 ^3, 6^	2.56 ± 0.72 2.05–3.07 ^5, 6^	0.23 ± 0.04 0.20–0.26 ^3, 6^	5.63 ± 0.56 5.23–6.03 ^4^
*Artemisia* *tridentate* Big sagebrush (*n* = 2)	0.49 ± 0.01 0.48–1.32 ^2^	0.09 ± 0.01 0.08–0.33 ^6^	0.22 ± 0.40 0.19–0.48 ^2^	0.51 ± 0.08 0.45–1.82 ^2^	0.53 ± 0.38 0.26–1.08 ^2^	1.55 ± 0.02 1.53–2.98 ^6^	0.04 ± 0.01 0.04–0.71 ^2, 6^	0.01 ± 0.00 0.01–0.13 ^2, 6^	0.26 ± 0.23 0.24–3.72 ^1^
*Artemisia* *tridentate* root Big sagebrush root (*n* = 2)	1.13 ± 0.27 0.95–1.32 ^5^	0.31 ± 0.18 0.30–0.33 ^3, 6^	0.48 ± 0.13 0.47–0.48 ^5^	1.66 ± 0.22 1.50–1.82 ^5^	0.99 ± 0.13 0.91–1.08 ^5^	2.67 ± 0.43 2.37–2.98 ^3, 6^	0.63 ± 0.12 0.54–0.71 ^5, 6^	0.12 ± 0.02 0.10–0.13 ^3, 6^	3.45 ± 0.39 3.17–3.72 ^4^
*Juniper monosperma* One–seed juniper (*n* = 2)	0.74 ± 0.03 0.49–1.32 ^2^	0.04 ± 0.17 0.03–0.61 ^6^	0.22 ± 0.01 0.21–0.47 ^2^	0.50 ± 0.02 0.49–0.91 ^2^	0.34 ± 0.03 0.26–1.08 ^2^	1.10 ± 0.68 1.03–1.17 ^6^	0.07 ± 0.01 0.06–0.20 ^2, 6^	0.02 ± 0.00 0.02–0.24 ^2, 6^	0.39 ± 0.01 0.38–2.08 ^1^
*Juniperus monosperma* One–seed juniper root (*n* = 2)	0.73 ± 0.15 0.62–0.84 ^5^	0.06 ± 0.01 0.05–0.06 ^3, 6^	0.38 ± 0.13 0.29–0.47 ^5^	0.85 ± 0.98 0.78–0.91 ^5^	0.60 ± 0.04 0.58–0.63 ^5^	1.09 ± 0.88 1.03–1.15 ^3, 6^	0.17 ± 0.04 0.15–0.20 ^5, 6^	0.22 ± 0.03 0.19–0.24 ^3, 6^	2.07 ± 0.13 2.07–2.08 ^4^
Soil range (*N* = 32)	0.87–5.20 ^2, 5^	0.04–0.18 ^3^	0.63–2.02 ^2, 5^	3.38–13.80 ^2, 5^	0.04–0.24 ^2, 5^	0.85–3.49 ^3^	1.41–8.61 ^2, 5^	0.21–1.46 ^2, 3^	5.24–15.10 ^1, 4^
WHO RMPPL [47]	1 mg/kg	0.3 mg/kg	*	10 mg/kg	*	*	*	*	*

Note: Soil compared to aboveground plant: ^1^ *p* < 0.01, ^2^ *p* < 0.001; Soil compared to root: ^3^ *p* < 0.05, ^4^ *p* < 0.01; ^5^ *p* < 0.001; Aboveground plant compared to root: ^6^ *p* < 0.05; * There are no World Health Organization (WHO) Raw Medicinal Plant Permissible Levels (RMPPL) [47] for Cs, Mo, Se, Th, U, and V.

**Table 3:** Similar plant and soil studies examining metal(loid) concentrations. Metal(loid) concentrations are reported as mg/kg from high-impact areas unless otherwise specified.

Sample Type and/or Scientific Name of Plant(s)	Metal(loid) Concentration (mg/kg)	Reference
*Agrimonia eupatoria, Anthyllis vulneraria, Artemisia absinthium, Centaurium erythraea, Chelidonium, majus, Cichorium intybus, Corlylus avellana, Echium vulgare, Epilobium parviflora, Equisetum arvense, Galium verum, Genista tinctoria, Geum urbanum, Hypericum perforatum, Leonurus cardiaca, Lycopus europaeus, Lyssimachia nummularia, Lythrum salicaria, Melilotus officinalis, Mentha longifolia, Mentha pulegium, Origanum vulgare, Potentilla anserina, Solidago virgaurea, Taraxacum officinale, Thymus pulegioides, Trifolium arvense, Urtica dioica, Valeriana officinalis, Verbena officinalis, Viola tricolor.*	V: 0.031–76.3 (aerial parts) V: 0.026–14.5 (leaves)	Antal et al. [49]
“Vegetation” [42] (p. 12)	U: 0.5–7.7/roots: 5.0/shoots: 2.4	de Lemos et al. [42]
*Sterculia setigera* Del., ^a^* Sclerocarya birrea* (A. rich.) Hochst)	Cd: 0.00 ± 0.00–0.21 ± 0.07 Pb: 0.63 ± 0.02–1.08 ± 0.07 leaves/stems ^*a*^	Isa et al. [50]
*Cassia senna (L.)* (Senna makki)*, Corchorus trilocularis* (Mundheri)*, Solanum indicum* (Brihat Kantakari)*, Mentha arvensis (L.)* (Podina)*, ^b^ Withania somnifera (L.)* (Dunal, Asgand)*, Xanthium strumariue (L.)* (chot gokhru)*, ^c^Asparagus rasemosus* willd (satavara, satawar)	Se: 1.64–2.26 (leaves) Se: 1.26–1.50 (roots)*^b^*	Kolachi et al. [51]
*^d^ Tilia cordata, Matricaria chamomilla, Calendula officinalis, Ocimum basilicum, Achillea millefolium, Hypericum perforatum*	Th: 10–60 mBq/kg Cs: < 60 mBq/kg *^c^* Pb: 10–30 mBq/kg U: 10–40 mBq/kg *^c^*	Oprea et al. [52]
*Thelesperma megapotamicum* Sprengel Kuntze	As: 0.42 ± 0.10 (h)/0.76 ± 0.24 (r)/1.84–2.32 (s) Cd: 0.35 ± 0.31 (h)/0.63 ± 0.66 (r)/0.05–0.51 (s) Cs: 0.06 ± 0.07 (h)/0.21 ± 0.17 (r)/0.49–1.08 (s) Pb: 0.30 ± 0.73 (h)/0.81 ± 0.29 (r)/5.21–5.78 (s) Mo: 7.92 ± 9.30 (h)/18.30 ± 21.56 (r)/nd–10.56 (s) Se: 0.74 ± 0.39 (h)/1.24 ± 0.56 (r)/nd–1.12 (s) Th: 0.20 ± 0.25 (h)/0.27 ± 0.16 (r)/2.64–2.94 (s) U: 0.02 ± 0.01 (h)/0.11 ± 0.04 (r)/0.83–1.29 (s) V: 0.24 ± 0.10 (h)/2.34 ± 1.59 (r)/9.20–16.30 (s)	Samuel-Nakamura et al. [43]
Soil	Se: 2.3/0.7 (control)	Dreesen and Cokal [56]
Soil	U: 3–8	deLemos et al. [42]
Soil	U: 5.1 ± 2.0/4.2 ± 2.0 (low–impact area)	deLemos et al. [57]

Note: ^*a*^ *Sclerocarya birrea* leaves and stems; ^*b*^ *Withania somnifera* (L.) roots; ^*c*^ *Asparagus rasemosus roots*; ^*d*^ *Cs concentration levels of Tilia cordata aerial parts/flowers*; h = herb; r = root; s = root/topsoil; nd = not detected.

**Table 4:** Provisional Tolerable Weekly Intake (PTWI) of As, Cd, and Pb through the ingestion of several species of medicinal plants.

Plant Species Scientific/Common Names	Metal(loid)	Weekly Intake (μg/kg BW)	PTWI (μg/kg BW)	% of PTWI
*Achnaterum hymenoides* Indian ricegrass	As Cd Pb	0.76 0.04 1.09	15 7 25	5.07 0.57 4.36
*Aristida purpurea* Purple threeawn	As Cd Pb	0.71 0.13 1.55	15 7 25	4.73 1.86 6.2
*Bouteloua gracilis* Blue grama	As Cd Pb	0.63 0.06 1.16	15 7 25	4.2 0.86 4.64
*Bouteloua gracilis* root Blue grama root	As Cd Pb	0.68 0.07 1.25	15 7 25	4.53 1 5.0
*Pascopyrum smithii* Western wheatgrass	As Cd Pb	0.69 0.05 1.13	15 7 25	4.6 0.71 4.52
*Pleuraphis jamesii* Galleta	As Cd Pb	0.82 0.51 1.25	15 7 25	5.47 7.3 5.0
*Sporobolus cryptandrus* Sand dropseed	As Cd Pb	0.62 0.11 1.17	15 7 25	4.13 1.6 4.68
*Artemisia tridentate* Big sagebrush	As Cd Pb	0.29 0.05 0.30	15 7 25	1.93 0.71 1.2
*Juniper monosperma* One-seed juniper	As Cd Pb	0.29 0.02 0.29	15 7 25	1.93 0.29 1.16

Note: BW = reference body weight (60 kg); there are no PTWI for Cs, Mo, Se, Th, U, or V.

**Table 5:** Reference Dietary Intake (RDI) or Recommended Dietary Allowance (RDA) and Upper Limit (UL) of Mo, Se, and V through the ingestion of several species of medicinal plants.

Plant Species Scientific/Common Names	Metal(loid)	Daily Intake (μg)	RDI or RDA and UL (μg/day)	% of RDI or RDA and UL
*Achnaterum hymenoides* Indian ricegrass	Mo Se V	0.61 1.10 3.57	RDA 45/UL 2000 RDI 55/UL 400 UL 1800	1.36/0.03 2.0/0.28 0.21
*Aristida purpurea* Purple threeawn	Mo Se V	0.64 1.93 4.86	RDA 45/UL 2000 RDI 55/UL 400 UL 1800	1.42/0.03 3.51/0.48 0.27
*Bouteloua gracilis* Blue grama	Mo Se V	0.64 1.47 1.29	RDA 45/UL 2000 RDI 55/UL 400 UL 1800	1.42/0.03 2.67/0.37 0.07
*Bouteloua gracilis* root Blue grama root	Mo Se V	0.50 1.50 2.93	RDA 45/UL 2000 RDI 55/UL 400 UL 1800	1.11/0.03 2.73/0.37 0.16
*Pascopyrum smithii* Western wheatgrass	Mo Se V	0.28 0.97 2.88	RDA 45/UL 2000 RDI 55/UL 400 UL 1800	0.62/0.01 1.76/0.24 0.16
*Pleuraphis jamesii* Galleta	Mo Se V	0.89 2.01 3.25	RDA 45/UL 2000 RDI 55/UL 400 UL 1800	1.98/0.04 3.65/0.50 0.18
*Sporobolus cryptandrus* Sand dropseed	Mo Se V	0.91 1.91 3.64	RDA 45/UL 2000 RDI 55/UL 400 UL 1800	2.02/0.05 3.47/0.48 0.20
*Artemisia tridentate* Big sagebrush	Mo Se V	0.44 1.29 0.22	RDA 45/UL 2000 RDI 55/UL 400 UL 1800	0.98/0.02 2.35/0.32 0.01
*Juniper monosperma* One-seed juniper	Mo Se V	0.29 0.92 0.32	RDA 45/UL 2000 RDI 55/UL 400 UL 1800	0.64/0.01 1.67/0.23 0.02

Note: There are no RDIs/RDAs or ULs for As, Cs, Pb, Th, or U.

## Data Availability

Restrictions apply to the availability of these data. Data was obtained from The Navajo Nation and are available with the permission from The Navajo Nation Human Research Review Board.

## References

[1] Amiri A., Zhao S. (2019). Environmental justice screening tools: Implications for nursing. Public Health Nurs..

[2] Mitchell F.M. (2019). Water (in)security and American Indian health: Social and environmental justice implications for policy, practice, and research. Public Health.

[3] Trask H. (1999). From a Native Daughter: Colonialism and Sovereignty in Hawaii.

[4] Lewis J., Hoover J., MacKenzie D. (2017). Mining and Environmental Health Disparities in Native American Communities. Curr. Environ. Health Rep..

[5] McLemore V.T. (1983). Uranium Industry in New Mexico—History, Production and Present Status.

[6] Brugge D., Panikkar B. (2007). The ethical issues in uranium mining research in the Navajo Nation. Acc. Res..

[7] Hoover J., Erdei E., Nash J., Gonzales M. (2019). A review of metal exposure studies conducted in the rural southwestern and mountain region of the United States. Curr. Epidemiol. Rep..

[8] Taylor D.M., Taylor S.K. (1997). Environmental uranium and human health. Rev. Environ. Health.

[9] Gilman A.P., Villenueve D.C., Secours V.E., Yagminas A.P., Tracy B.L., Quinn J.M., Valli V.E., Willes R.J., Moss M.A. (1998). Uranyl nitrate: 28-day and 91-day toxicity studies in the Sprague-Dawley rat. Toxicol. Sci..

[10] Hoover J., Gonzales M., Shuey C., Barney Y., Lewis J. (2017). Elevated arsenic and uranium contamination in unregulated water sources on the Navajo Nation, USA. Expo. Health.

[11] Eisler R. (1988). Arsenic hazards to fish, wildlife, and invertebrates: A synoptic view. US Fish. Wildl. Serv. Biol. Rep..

[12] Fan X.T. (2016). Effects of Soil Amendments on Growth, Metabolism and Cadmium Uptake by Panax Notoginseng.

[13] Han R., Zhou B., Huang Y., Lu X., Li S., Li N. (2020). Bibliometric overview of research trends on heavy metal health risks and impacts in 1989–2018. J. Clean. Prod..

[14] Mikulski M.A., Wichman M.D., Simmons D.L., Pham A.N., Clottey V., Fuortes L.J. (2017). Toxic metals in ayurvedic preparations from a public health lead poisoning cluster investigation. Int. J. Occup. Environ. Health.

[15] Kumkrong P., LeBlanc K.L., Mercier P.H., Mester Z. (2018). Selenium analysis in waters. Part 1: Regulations and standard methods. Sci. Total Environ..

[16] Meeker J.D., Rossano M.G., Protas B., Diamond M.P., Puscheck E., Daly D., Paneth N., Wirth J.J. (2008). Cadmium, lead, and other metals in relation to semen quality: Human evidence for molybdenum as a male reproductive toxicant. Environ. Health Perspect..

[17] Meeker J.D., Rossano M.G., Protas B., Padmanahban V., Diamond M.P., Puscheck E., Daly D., Paneth N., Wirth J.J. (2010). Environmental exposure to metals and male reproductive hormones: Circulating testosterone is inversely associated with blood molybdenum. Fertil. Steril..

[18] Yang F., Yi X., Guo J., Shuaishuai X., Xiao Y., Huang X., Duan Y., Luo D., Xiao S., Huang Z. (2019). Association of plasma and urine metals levels with kidney function: A population-based cross-sectional study in China. Chemosphere.

[19] International Agency for Research on Cancer (IARC, 2012) Working Group on the Evaluation of Carcinogenic Risks to Humans. Arsenic, Metals, Fibres and Dusts. Lyon (FR): International Agency for Research on Cancer; 2012. (IARC Monographs on the Evaluation of Carcinogenic Risks to Humans, No. 100C.). https://www.ncbi.nlm.nih.gov/books/NBK304375/.

[20] Pyrzynska K. (2020). Nanomaterials in speciation analysis of metals and metalloids. Talanta.

[21] Yu H.-S., Liao W.-T., Chai C.-Y. (2006). Arsenic carcinogenesis in the skin. J. Biomed. Sci..

[22] International Agency for Research on Cancer (IARC, 2006) IARC Monographs on the Evaluation of Carcinogenic Risks to Humans Volume 87, Inorganic and Organic Lead Compounds. https://publications.iarc.fr/Book-And-Report-Series/Iarc-Monographs-On-The-Identification-Of-Carcinogenic-Hazards-To-Humans/Inorganic-And-Organic-Lead-Compounds-2006.

[23] Charen E., Harbord N. (2020). Toxicity of herbs, vitamins, and supplements. Adv. Chronic Kidney Dis..

[24] Rahman I.U., Afzal A., Iqbal Z., Ijaz F., Ali N., Shah M., Ullah S., Bussmann R.W. (2019). Historical perspectives o ethnobotany. Clin. Dermatol..

[25] Dunmire W.W., Tierney G.D. (1997). Wild Plants and Native Peoples of the Four Corners.

[26] Hosen N., Nakamura H., Hamzah A. (2020). Adaptation to climate change: Does traditional ecological knowledge hold the key?. Sustainability.

[27] Tsuji L.J.S., Manson H., Wainman B.C., Vanspronsen E.P., Shecapio-Blacksmith J., Rabbitskin T. (2007). Identifying potential receptors and routes of contaminant exposure in the traditional territory of the Ouje-Bougoumou Cree: Land use and a geographical information system. Environ. Monit. Assess..

[28] Mayes V.O., Lacy B.B. (2012). Nanise’ A Navajo Herbal One Hundred Plants from the Navajo Reservation.

[29] United States Department of Agriculture (USDA, 2000) Indian Ricegrass Plant Guide. https://plants.usda.gov/DocumentLibrary/plantguide/pdf/pg_achy.pdf.

[30] Arkush B.S., Arkush D. (2021). Aboriginal plant use in the central Rocky Mountains: Macrobotanical records from three prehistoric sites in Birch Creek Valley, eastern Idaho. N. Am. Archaeol..

[31] United States Department of Agriculture (USDA, 2020) Purple Threeawn Plant Guide. https://plants.usda.gov/DocumentLibrary/plantguide/pdf/pg_arpu9.pdf.

[32] United States Department of Agriculture (USDA, 2002) Big Sagebrush Plant Fact Sheet. https://plants.usda.gov/DocumentLibrary/factsheet/pdf/fs_artr2.pdf.

[33] Shemluck M. (1982). Medicinal and other uses of the compositae by Indians in the United States and Canada. J. Ethnopharmacol..

[34] United States Department of Agriculture (USDA, 2007) Blue Grama Plant Guide. https://plants.usda.gov/home/plantProfile?symbol=BOGR2.

[35] United States Department of Agriculture (USDA, 2014) Plants. *Juniperus monosperma* (Englm). Sarg. General. Website: United States Department of Agriculture (USDA, 2014). https://www.fs.fed.us/database/feis/plants/tree/junmon/all.html.

[36] United States Department of Agriculture (USDA, 2000) Western Wheatgrass Plant Guide. https://plants.usda.gov/DocumentLibrary/plantguide/pdf/pg_pasm.pdf.

[37] United States Department of Agriculture (USDA, 2012) James’ Galleta Plant Guide. https://plants.usda.gov/DocumentLibrary/plantguide/pdf/pg_plja.pdf.

[38] Matthews W. (1886). Navajo names for plants. Am. Nat..

[39] United States Department of Agriculture (USDA, 2010) Sand Dropseed Plant guide. https://plants.usda.gov/DocumentLibrary/plantguide/pdf/pg_spcr.pdf.

[40] Hocking G.M. (1956). Some plant materials used medicinally and otherwise by the Navaho Indians in the Chaco Canyon, New Mexico. El Palacio.

[41] Vestal P.A. (1952). Ethnobotony of the Ramah Navajo. Papers of the Peabody Museum of American Archeology and Ethnology.

[42] de Lemos J.L., Brugge D., Cajero M., Downs M., Durant J.L., George C.M., Henio-Adeky S., Nez T., Manning T., Rock T. (2009). Development of risk maps to minimize uranium exposures in the Navajo Churchrock mining district. Environ. Health.

[43] Samuel-Nakamura C., Hodge F.S. (2019). Occurrence and risk of meta(loid)s in *Thelesperma megapotamicum*. Plants.

[44] Fromberg E., Goble R., Sanchez V., Quigley D. (2000). The assessment of radiation exposure in Native American communities from nuclear weapons testing in Nevada. Soc. Risk Analysis.

[45] Wolfley J. (1998). Ecological risk assessment and management: Their failure to value Indigenous traditional ecological knowledge and protect tribal homelands. Am. Indian Cult. Res. J..

[46] Harmon M.E., Lewis J., Miller C., Hoover J., Shuey C., Cajero M., Lucas S., Zychowski K., Pacheco B., Erdie E. (2017). Residential proximity to abandoned uranium mines and serum inflammatory potential in chronically exposed Navajo communities. J. Expo. Sci. Environ. Epidemiol..

[47] World Health Organization (1998). Quality Control Methods for Medicinal Plant Materials.

[48] Samuel-Nakamura C., Hodge F.S., Sokolow S., Ali A.S., Robbins W.A. (2019). Metal(loid)s in *Cucurbita pepo* in a Uranium Mining Impacted Area in Northwestern New Mexico, USA. Int. J. Environ. Res. Public Health.

[49] Antal D., Dehelean C., Dobrea C., Manfred A. (2009). Vanadium in medicinal plants: New data on the occurrence of an element both essential and toxic to plants and man. An. Univ. Din Oradea Fasc. Biol..

[50] Isa P.A., Hitler L., Joseph I.A., Oyetola O. (2017). Evaluation of heavy metals concentrations in selected medicinal plants (*Sterculia setigera* Del. And *Sclerocarya birrea* (A. rich.) Hochst) collected from Bwabul Spring, Bambuka and Jalingo Low-lands, Traba State. J. Environ. Anal. Chem..

[51] Kolachi N.F., Kazi T.G., Afridi H.I., Khan S., Wadhwa S.K., Shah A.Q., Shah F., Baig J.A., Sirajuddin (2010). Determination of selenium content in aqueous extract of medicinal plants used as herbal supplement for cancer patients. Food. Chem. Toxicol..

[52] Oprea E.M., Pintilie V., Bufnea V., Aprotosoaie A.C., Cioanca O., Trifan A., Hancianu M. (2014). Radionuclides content in some medicinal plants commonly used in Romania. Farmacia.

[53] Shi Y.-z., Ruan J.-y., Ma L.-f., Han W.-y., Wang F. (2008). Accumulation and distribution of arsenic and cadmium by tea plants. J. Zhejiang Univ. Sci. B.

[54] Anke M., Seeber O., Muller R., Schafer U., Zerull J. (2009). Uranium transfer in the food chain from soil to plants, animals, and man. Chemie Erde-Geochem..

[55] Soudek P., Petrova S., Benesova D., Dvorakova M., Vanek T. (2011). Uranium uptake by hydroponically cultivated crop plants. J. Environ. Radioact..

[56] Dreesen D.R., Cokal E.J. (1984). Plant uptake assay to determine bioavailability of inorganic contaminants. Water Air Soil Pollut..

[57] deLemos J.L., Bostick B.C., Quicksall A.N., Landis J.D., George C.C., Slagowski N.L., Rock T., Brugge D., Lewis J., Durant J.L. (2008). Rapid dissolution of soluble uranyl phases in arid, mine-impacted catchments near Church Rock, NM. Environ. Sci. Technol..

[58] Wu J., Zou Y., Zhan X., Chen S., Lu G., Lai F. (2008). Survey of heavy metal pollution in four Chinese crude drugs and their cultivated soils. Bull. Environ. Contam. Toxicol..

[59] Barthwal J., Nair S., Kakkar P. (2008). Heavy metal accumulation in medicinal plants collected from environmentally different sites. Biomed. Environ. Sci..

[60] Sarma H., Deka S., Deka H., Saikia R.R. (2011). Accumulation of heavy metals in selected medicinal plants. Rev. Environ. Contam. Toxicol..

[61] Arpadjan S., Celik G., Taskesen S., Gucer S. (2008). Arsenic, cadmium and lead in medicinal herbs and their fractionation. Food Chem. Toxicol..

[62] Naithani V., Kakkar P. (2006). Effect of ecological variation on heavy metal content of some medicinal plants used as herbal tea ingredients in India. Bull. Environ. Contam. Toxicol..

[63] Joint Expert Committee on Food Additives (JECFA) (1989). Evaluation of Certain Food Additives and Contaminants. Thirty-Third Report of the Joint Food and Agriculture Organization (FAO)/World Health Organization (WHO) Expert Committee on Food Additives.

[64] Joint Expert Committee on Food Additives (JECFA) (2003). Joint FAO/WHO Expert Committee on food additives. Sixty-First Meeting: Summary and Conclusions.

[65] Food and Nutrition Board (2001). Dietary Reference Intakes for Vitamin A, Vitamin K, Arsenic, Boron, Chromium, Copper, Iodine, Iron, Manganese, Molybdenum, Nickel, Silicon, Vanadium, and Zinc.

[66] Food and Nutrition Board (2000). Dietary Reference Intakes for Vitamin C, Vitamin E, Selenium and Carotenoids.

[67] World Health Organization (2007). WHO Guidelines for Assessing Quality of Herbal Medicines with Reference to Contaminants and Residues.

[68] Harmon M.E., Campen M.J., Miller C., Shuey C., Cajero M., Lucas S., Pacheco B., Erdei E., Ramone S., Nez T. (2016). Associations of circulating oxidized LDL and conventional biomarkers of cardiovascular disease in a cross-sectional study of the Navajo population. PLoS ONE.

[69] Salahinejad M., Aflaki F. (2010). Toxic and essential mineral elements content of black tea leaves and their tea infusions consumed in Iran. Biol. Trace Elem. Res..

[70] Navarro-Alarcon M., Cabrera-Vique C. (2008). Selenium in food and the human body: A review. Sci. Total Environ..

[71] Yuan C., Gao E., He B., Jiang G. (2007). Arsenic species and leaching characteristics in tea (*Camellia sinensis*). Food Chem. Toxicol..

[72] Basgel S., Erdemoglu S.B. (2006). Determination of mineral and trace elements in some medicinal herbs and their infusions consumed in Turkey. Sci. Total Environ..

[73] Navajo Nation Division of Natural Resources (NNDNR) (2008). Navajo Endangered Species List.

[74] Forestry Division, New Mexico Energy, Minerals, and Natural Resources Department (NMEMNRD) (2017). New Mexico Rare Plant. Conservation Strategy.

